# Intradermal administration of fractional doses of the inactivated poliovirus vaccine in a campaign: a pragmatic, open-label, non-inferiority trial in The Gambia

**DOI:** 10.1016/S2214-109X(21)00497-6

**Published:** 2021-12-21

**Authors:** Adedapo O Bashorun, Mariama Badjie Hydara, Ikechukwu Adigweme, Ama Umesi, Baba Danso, Njilan Johnson, Ngally Aboubacarr Sambou, Sidat Fofana, Francis J Kanu, Visalakshi Jeyaseelan, Harish Verma, William C Weldon, M Steven Oberste, Roland W Sutter, David Jeffries, Miriam Wathuo, Ondrej Mach, Ed Clarke

**Affiliations:** a Medical Research Council Unit, The Gambia at the London School of Hygiene and Tropical Medicine, Banjul, The Gambia; b Ministry of Health, Government of The Gambia, Banjul, The Gambia; c World Health Organization, Atlanta, GA, USA; d Centers for Disease Control and Prevention, Geneva, Switzerland

## Abstract

**Background:**

A rapid increase in circulating vaccine-derived poliovirus type 2 outbreaks, and the need to reserve inactivated poliovirus vaccine (IPV) for routine immunisation, has increased the value of fractional dose IPV (fIPV) as a measure to prevent acute flaccid paralysis. However, the intradermal route of administration has been viewed as prohibitive to outbreak response campaigns. We aimed to establish the immunogenicity and safety of administering intradermal fIPV with a disposable syringe jet injector (DSJI) or an intradermal adaptor (IDA) compared with standard administration with a BCG needle and syringe (N&S).

**Methods:**

This pragmatic, non-inferiority trial was undertaken in a campaign setting in communities in The Gambia. Children aged 4–59 months without contraindication to vaccination were eligible. Children were not individually randomly assigned; instead, the vaccination teams were randomly assigned (1:1:1) to one of three administration methods. Parents and the field team were not masked, but laboratory personnel were masked. Baseline demographic and anthropometric data were collected from the participants. Public health officers experienced at intradermal immunisation, and nurses without experience, had 2 h of training on each of the administration methods before the campaign. Participants were vaccinated using the administration method in use by the vaccination team in their community. Poliovirus serum neutralising antibodies (SNA) were measured in children aged 24–59 months before and 4 weeks after vaccination. Adverse events and data on injection quality were collected from all participants. The primary outcome was the type 2 immune response rate (seroconversion in seronegative [SNA titre <8] children plus a 4-fold titre rise in seropositive children). Adjusted differences in the immune response between the DSJI or IDA group versus the N&S group were calculated with 97·5% CIs. A margin of −10% was used to define the non-inferiority of DSJI or IDA compared to N&S. Immunogenicity analysis was done per protocol. The trial is registered with ClinicalTrials.govNCT02967783 and has been completed.

**Findings:**

Between Oct 28 and Dec 29, 2016, 3189 children aged 4–59 months were recruited, of whom 3170 were eligible. Over 3 days, 2720 children were vaccinated (N&S, 917; IDA, 874; and DSJI, 929). Among 992 children aged 25–59 months with a baseline SNA available, 90·1% (95% CI 86·1–92·9; 281/312) of those vaccinated using the DSJI had an immune response to type 2 compared with 93·8% (90·6–95·8; 331/353) of those vaccinated with N&S and 96·6% (94·0–98·0; 316/327) of those vaccinated with IDA. All (53/53) type 2 seronegative children seroconverted. For polio type 2, non-inferiority was shown for both the IDA (adjusted difference 0·7% [97·5% CI −3·3 to 4·7], unadjusted difference 2·9% [–0·9 to 6·8]) and DSJI (adjusted difference −3·3% [–8·3 to 1·5], unadjusted difference −3·7% [–8·7 to 1·1]) compared with N&S. Non-inferiority was shown for type 1 and 3 for the IDA and DSJI. Neither injection quality nor the training and experience of the vaccinators had an effect on immune response. No safety concerns were reported.

**Interpretation:**

In a campaign, intradermal fIPV is safe and generates consistent immune responses that are not dependent on vaccinator experience or injection quality when administered using an N&S, DSJI, or IDA. Countries facing vaccine-derived poliovirus type 2 outbreaks should consider fIPV campaigns to boost population immunity and prevent cases of acute flaccid paralysis.

**Funding:**

World Health Organization and the Medical Research Council.

## Introduction

Although the goal of global polio eradication is seemingly within reach, there are still important hurdles to overcome.^1^, ^2^ Wild poliovirus type 2 has not been detected worldwide since 1999, and type 3 since 2012, and these strains were declared to have been eradicated in 2015 (for type 2) and 2019 (for type 3).^3^ Furthermore, WHO's African Region was certified as being free of wild polioviruses on Aug 25, 2020.

**Research in context**

**Evidence before this study**

A PubMed search to identify articles published before July 31, 2021, was conducted using the following search terms with appropriate Boolean operators: “inactivated poliovirus vaccine”, “intradermal”, “vaccine derived poliovirus”, “campaign”, “pragmatic”, “meta-analysis”, “systematic review”, “randomized controlled trial”, “clinical trial”, “immunogenicity”, and “safety”. There were no language restrictions. Two meta-analyses published in 2019 and 2021, albeit including only one trial from a low-income country, which was conducted in The Gambia, have compared equivalent full-dose and fractional-dose inactivated poliovirus vaccine (IPV) schedules. The seroconversion rates after a single fractional dose of IPV delivered by the intradermal route were lower than the seroconversion rates generated by a full intramuscular dose of the vaccine. Any difference in seroconversion after the second and third doses are progressively less than that after the first dose. Median antibody titres are consistently lower after fractional dose schedules than full-dose schedules. There are no definitive trials comparing needle and syringe (N&S) with intradermal adaptor (IDA) or the disposable syringe jet injector (DSJI) for intradermal fractional IPV (fIPV) dose administration. Nonetheless, two fIPV doses are more immunogenic that a single full dose of the vaccine as well as being dose sparing. In addition, intradermal fIPV boosts mucosal immunity in those previously primed with oral poliovirus vaccine in the same way as an intramuscular dose. Given progressive increases in circulating vaccine-derived poliovirus type 2 (cVDPV2) outbreaks, waning type 2 population immunity, and the need to reserve IPV for routine immunisations, data to support country decisions regarding the use of intradermal fIPV in outbreak campaigns, particularly in sub-Saharan Africa, are needed.

**Added value of this study**

Fractional doses of IPV can be delivered reliably by the intradermal route using processes and personnel closely aligned to those used during a community outbreak response campaign in rural west Africa. The immune responses generated against poliovirus type 2 as well as against the type 1 and 3 viruses are similar irrespective of whether the vaccine is administered by an N&S, a DSJI, or using an IDA. These responses are not substantially altered by injection quality and are independent of the amount of previous experience the vaccinator has at giving intradermal injections. Intradermal immunisation in the community is safe and well tolerated.

**Implications of all the available evidence**

Strong data are available to support the use of intradermal fIPV in community campaigns for cVDPV2 outbreaks, including in rural sub-Saharan Africa and in settings with high amounts of malnutrition, and also to support its use in campaigns designed to address the immunity gap in under-immunised populations. Countries facing cVDPV2 outbreaks should be encouraged to grasp the opportunity intradermal fIPV campaigns provide to prevent avoidable paralytic disease in this context.

Both the oral poliovirus vaccine (OPV) and the inactivated poliovirus vaccine (IPV) are essential to the Global Polio Eradication Initiative's endgame strategy.^2^, ^4^ OPV induces systemic antibodies, protecting the individual from paralytic disease, but it also generates mucosal immunity, preventing the long-term excretion of the virus in the stool, and hence community transmission.^5^ However, two key disadvantages of OPV are the occurrence, albeit rarely, of vaccine-associated paralytic poliomyelitis and the emergence of circulating vaccine-derived polioviruses (cVDPV) that are genetically divergent from the parent vaccine strain, are associated with person-to-person transmission, and can also cause paralytic disease.^6^, ^7^ Although IPV induces a systemic antibody response, it induces little or no mucosal immunity in those who have not previously received OPV.^5^ In contrast, several studies have now shown that IPV boosts mucosal immunity more effectively than additional doses of OPV in those who have previously received OPV.^8^, ^9^, ^10^

In April, 2016, a switch from the use of trivalent to bivalent OPV, containing only the Sabin type 1 and type 3 strains, occurred worldwide. The switch aimed to reduce the occurrence of cVDPV, of which more than 85% of cases were attributable to the type 2 vaccine virus at the time.^2^, ^6^ However, although there were fewer than 100 cVDPV2 cases in up to five different countries in 2017 and 2018, there has subsequently been a sustained increase. More than 350 cases across 16 countries were detected in 2019, while more than 1000 cases across 24 countries were detected in 2020.^11^ Given the necessity to block community transmission, monovalent OPV type 2 campaigns have been the only available method of outbreak response. Consequently, although early outbreaks were largely seeded from trivalent OPV use before the switch, sequencing data confirm that new outbreaks have arisen from the use of monovalent OPV type 2.^1^

To maintain individual protection from type 2 paralytic disease, the switch was supposed to be accompanied by the introduction of a dose of IPV into the schedule of all countries using only OPV. However, because of supply constraints, many countries were unable to introduce IPV or had vaccine stockouts. When combined with poor routine immunisation coverage in many countries, this has resulted in an estimated 143 million children across serial cohorts born since 2016, most of them in sub-Saharan Africa, who are yet to receive IPV and therefore do not have any vaccine-induced immunity against poliovirus type 2.^1^, ^12^

The provision of IPV to mitigate the risk of paralysis in cVDPV2 outbreaks, and for catch-up campaigns designed to fill the immunity gaps in non-immunised populations, continues to be limited by the need to prioritise doses for routine immunisation.^13^ However, considerable data exist to support the use of fractional (a fifth; 0·1 mL) IPV (fIPV) doses delivered by the intradermal route. The WHO Strategic Advisory Group of Experts on Immunization recommended that countries consider using two fIPV doses for routine immunisation as well as for outbreak response campaigns.^14^ This schedule is more immunogenic than a single full dose of IPV, in addition to being dose-sparing.^15^, ^16^ In OPV-immunised individuals, fIPV also boosts mucosal immunity to a similar degree to full-dose IPV, making it suitable for cVDPV2 outbreaks.^10^, ^17^

A key concern with the use of intradermal fIPV in campaigns is the feasibility of delivering intradermal injections in the community on a large scale. Public health personnel across much of sub-Saharan Africa routinely give the BCG vaccine by the intradermal route. However, given the scale of the vaccination campaigns, the use of additional personnel, generally with little or no experience of giving intradermal injections, is essential for their success. Although OPV can be given reliably after minimal training, the need to deliver IPV intradermally rather than orally has been viewed as prohibitive to scale up. Several needle-free devices and other devices designed to facilitate intradermal vaccine delivery have been developed and assessed in clinical trials, producing generally supportive results.^18^ However, how these finding translate when intradermal immunisations need to be given rapidly as part of an outbreak response campaign is unknown.

This pragmatic trial aimed to determine the non-inferiority (in terms of immunogenicity) as well as the safety of administering intradermal fIPV with a disposable syringe jet injector (DSJI) or an intradermal adaptor (IDA) compared with standard BCG needle and syringe (N&S)-based administration using processes and personnel normally employed to deliver vaccination campaigns with injectable vaccines in The Gambia.

## Methods

### Study design and participants

This was an open-label, non-inferiority trial. The trial was pragmatic in design, meaning it aimed to emulate, as closely as possible, previous campaigns with parenteral vaccines undertaken in The Gambia.^19^ Therefore, public health officers, who are normally responsible for the conduct of such campaigns, were involved throughout the planning and implementation of the study. The trial was approved by The Gambia Government/Medical Research Council Joint Ethics Committee, and the WHO Research Ethics Review Committee. Clinical trial authorisation was obtained from The Gambian Medicines Control Agency. The trial was conducted according to the International Council for Harmonization Good Clinical Practice guidelines.

Widespread community sensitisation was undertaken across a rural setting in the western region of The Gambia to inform families of the planned study (appendix p 1). Families with children aged 4–59 months, the target group for IPV campaigns, were subsequently invited to central points in their community (eg, a school or clinic). Once written informed consent was obtained, demographic information (sex, ethnic group, maternal schooling, and maternal occupation), polio vaccination history, and anthropometric data (weight and height) were collected on paper case report forms for subsequent entry into a validated OpenClinica clinical trial database. A 2·0 mL blood sample was collected from children aged 24–59 months. This age group had received trivalent OPV as part of their routine immunisations before the switch but had not received IPV (appendix p 4). In keeping with procedures for campaigns, all children were eligible unless they had a contraindication to vaccination (ie, previous anaphylaxis).

Both public health officers, who administer all BCG vaccines to newborn babies in The Gambia and hence were experienced at administering intradermal injections, and nurses, who had little or no previous experience, took part as vaccinators in the campaign. This approach reflects widespread practice during national campaigns with injectable vaccines, given an insufficient number of public health officers to achieve national coverage within the prescribed timeframe. The similarity of the intradermal injection experience between individual public health officers and between individual nurses was confirmed on the basis of their professional training and employment history as documented in their curriculum vitae and confirmed verbally. Neither the public health officers nor the nurses had used the DSJI or the IDA previously.

3 days before the campaign, all vaccinators received up to 2 h of training on each of the three administration methods (N&S, DSJI, and IDA). This training included having each method explained and shown, followed by a period of hands-on practice. The vaccinators were required to confirm they felt confident in their ability to use each method independently at the end of the training. Six vaccination teams, each of which included one public health officer and one nurse, were then allocated to one of the central points in their community of the type used in past campaigns (market areas, schools, and health clinics) across the study area where the vaccination points were set up.

### Randomisation and masking

To effectively replicate the flow of vaccinees during campaigns, children were not individually randomly assigned. Instead, the vaccination teams were randomly assigned (1:1:1) to either the N&S, DSIJ, or IDA group, to establish the administration method they would use on each day of the campaign (appendix p 2). Randomisation, based on a sequence generated by a statistician not otherwise involved in the study, was undertaken using opaque, sealed, tamper-evident envelopes only after the team make-up had been defined, training had been completed, and the geographical areas and vaccination points to be covered by each team had been decided. Parents were subsequently asked to attend the vaccination point most convenient for them during the 3-day campaign. They did not know in advance which administration method was being used but were not masked at the time the vaccination occurred. The field team assessing safety endpoints were not masked. Laboratory personnel assessing serological endpoints were masked.

### Procedures

All children received a single 0·1 mL dose (a fifth of a full dose) of IPV (Sanofi-Pasteur; Lyon, France) intradermally using one of three administration methods: by a 27 G × 10 mm fixed-needle, auto-disable N&S (Helm Medical, Hamburg, Germany); by an IDA (West Pharmaceutical Services, Eschweiler, Germany) in combination with a 27 G × 13 mm fixed-needle, auto-disable N&S (Helm Medical); or by a DSJI (Tropis; Pharmajet Golden, CO, USA; appendix p 3). A 0·5 mL dose of IPV contains IPV type 1 (Mahoney strain, 40 D-antigen units), type 2 (MEF-1 strain, 8 D-antigen units), and type 3 (Saukett strain, 32 D-antigen units).

Children were visited at home by trained field workers 3 days after vaccination and solicited injection-site (tenderness, erythema, and induration) and systemic (axillary temperature, vomiting, diarrhoea, reduced feeding, drowsiness, and irritability) adverse events data were collected and graded for severity according to protocol-defined criteria (appendix p 5). Unsolicited adverse events, including serious adverse events (hospitalisations, deaths, life-threatening events, and events resulting in persistent incapacity), were recorded for 4 weeks after vaccination by asking parents to attend a study clinic in their community if their child had any health complaints. Unsolicited adverse events were graded for severity and relatedness to study vaccination. During the campaign, data on the size of the intradermal fluid bleb, fluid loss onto the skin, time taken to administer each vaccine, and the amount of distress apparent in the child were collected, as previously described.^20^, ^21^ The number of fIPV doses obtained from each vial according to administration method was calculated to assess vaccine waste.

Children aged 24–59 months also had a follow-up 2·0 mL blood sample collected 4 weeks after vaccination. This sample, and the one taken at baseline, were used to assess serum neutralising antibodies (SNA) titres against poliovirus types 1, 2, and 3 according to established protocols at the Centers for Disease Control and Prevention (Atlanta, GA, USA).^22^ SNA titres, estimated using the Spearman-Kärber method, were reported as the reciprocal of the calculated 50% endpoint titre and reported to a maximum titre of 1448 or higher, which is the upper limit of quantification for the assay.

### Outcomes

The primary immunogenicity outcome was the immune response to poliovirus type 2 generated after intradermal fIPV administration with either the DSJI or the IDA compared with the reference N&S. The percentage of children with a SNA titre of 8 or more in their baseline or post-vaccination blood samples defined the seroprevalence at these points in the trial.^23^ Seroconversion was defined as a baseline SNA titre of less than 8 and a post-vaccination titre of 8 or more. Among the children who had a baseline SNA titre of 8–362, the percentage who had a four-fold rise in their SNA titre in the post-vaccination sample was also established. The percentage of children who had an immune response to the vaccine was calculated by combining the percentage of children who seroconverted with those who had a four-fold rise in SNA titres. Children with a baseline titre of more than 362 were excluded because a four-fold rise in SNA titres would have been beyond the upper limit of quantification for the assay.

Immune responses to poliovirus types 1 and 3 represented secondary immunogenicity objectives. Safety outcomes were the number and severity of solicited injection site and systemic adverse events on day 3 post-vaccination; the occurrence, severity, and relatedness of unsolicited adverse events; and serious adverse events in the 4 weeks after vaccination. An injection was defined to be of good quality if the fluid loss onto the skin was less than 10 uL and the bleb size was 5 mm or more.^21^ Additional qualitative data on vaccinator and parental experience was collected and will be reported separately.

### Statistical analysis

The immunogenicity analysis was done per protocol. This included all participants who received a vaccine during the campaign, had pre-vaccination and post-vaccination serological results available, and had no protocol deviations expected to affect the serological endpoints. Newcombe CIs (ie, a modified Wilson score for the difference between two proportions) were calculated for the difference between two immune response proportions.^24^ Non-inferiority of the difference between the percentage of children with an immune responses in the DSJI group (IR_DSJI_) or the IDA group (IR_IDA_) each compared with reference N&S group (IR_N&S_) was declared if the lower limit of the two-sided 97·5% Newcombe CI (Bonferroni correction to allow for a multiplicity of 2; IR_DSJI_–IR_N&S_ or IR_IDA_–IR_N&S_) was more than the −10% non-inferiority margin. The non-inferiority margin was defined based on the predicted public health effect of such a reduction compared with the potential benefits of the alternative administration methods. Given that individuals were not individually randomly assigned, stratified CIs were calculated to account for the baseline differences between groups. The CIs were stratified by age and sex, and for variables significantly associated (p value <0·2) with the immune response via multilevel logistic regression, described later, separately for each serotype. The stratified Newcombe CIs were combined, using continuity-corrected inverse variance weights,^25^ resulting in adjusted CIs for each immune response non-inferiority comparison and serotype.

A sample size of 510 per administration method provided 80% power with an α of 2·5% to independently declare either administration method (DSJI or IDA) non-inferior to the N&S. The sample size was calculated from a simulation of the design assuming an immune response rate of 64% in each group based on previous data from The Gambia^26^ and allowed for 15% of those sampled to be excluded. The sample size was not adjusted for potential clustering at the vaccination team level because the clusters were not known a priori and, given that the experience and training of the teams was standardised, we expected it to be small. Instead, the maximum number of participants that was feasible beyond the minimum, unadjusted sample size, were vaccinated over the 3-day campaign.

Multilevel logistic regression models were fitted to identify the factors associated with the immune response. A univariable analysis was done to establish the unadjusted association between each variable and the immune response. All factors were then fitted into a multivariable model and a backwards elimination procedure was performed until the final model had only variables with an overall p value of <0·2. This analysis was done separately for each serotype and also in a combined analysis for all serotypes. Intraclass correlation coefficients were calculated to quantify any effect of clustering within the teams on the responses generated, and also to examine the correlation of responses to the three serotypes within individuals.

Binomial exact CIs were calculated around the median antibody titres. Safety and other data were summarised descriptively.^27^ Statistical analysis was done in Stata version 13.1. The trial is registered with ClinicalTrials.gov NCT02967783 and has been completed. The WHO polio data safety monitoring board oversaw the study.

### Role of the funding source

The trial was funded by WHO through a grant from Rotary International and by the Medical Research Council (UK). The costs of the serological analysis were met by the Centers for Disease Control and Prevention. WHO personnel participated in the study design, data interpretation, and decision to submit for publication.

## Results

**Figure 1:**
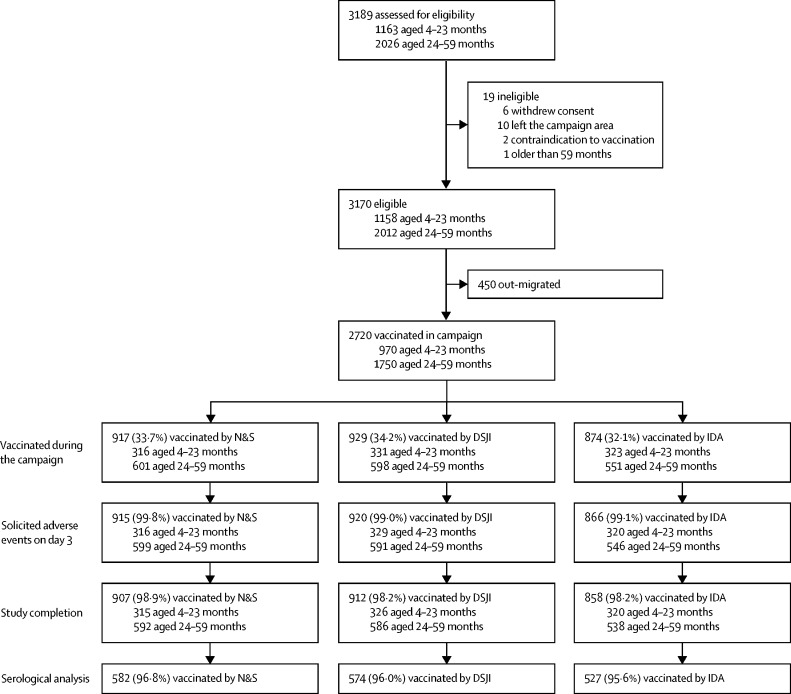
Trial profile Figure illustrating the number of infants and children who took part in the campaign separated into those aged 4–23 months and 24–59 months. Serological analysis (baseline and post-vaccination serum neutralising antibody titres for poliovirus types 1, 2, and 3) was only undertaken in those aged 24–59 months. DSJI=disposable syringe jet injector. IDA=intradermal adapter. N&S=needle and syringe.

Between Oct 28 and Dec 29, 2016, 3189 children aged between 4 and 59 months provided the baseline data for the study, of whom 3170 (99·4%) were eligible to take part in the campaign (figure 1). A period of political instability in The Gambia after baseline data collection resulted in substantial out-migration from the campaign area and delayed the campaign, which subsequently took place between Feb 7 and 9, 2017. At this point, 450 children (14·2%) had not returned to their previous place of residence and did not take part in the campaign. A total of 2720 children were vaccinated during the campaign over 3 days (N&S, 917 [33·7%]; DSJI, 929 [34·2%]; and IDA, 874 [32·1%]). Of these, 2701 (99·3%) had day 3 solicited reactogenicity collected, and 2677 (98·4%) completed the 4 four-week safety follow-up. Of the 1750 children aged 24–59 months, 1683 (96·2%) had a baseline and post-vaccination serological result available.

The baseline characteristics of all children vaccinated during the campaign are provided in table 1, and of those aged 24–59 months are provided in the appendix (p 6). Overall, just under two-thirds of children (1750 [64·3%] of 2720) were aged 24–59 months, 1366 (50·2%) were male, and 1354 (49·8%) were female. A fifth of children were stunted (height for age *Z* score <–2SD; 545 [20·0%]) and 196 (7·2%) were wasted (weight for height *Z* score <–2SD).

**Table 1:** Baseline demographic and anthropometric characteristics in all participants vaccinated during the campaign

		**Needle and syringe (n=917)**	**Disposable syringe jet injector (n=929)**	**Intradermal adapter (n=874)**	**Total (n=2720)**
Age (months)					
	4–23 months	316 (34·5%)	331 (35·6%)	323 (37·0%)	970 (35·7%)
	24–59 months	601 (65·5%)	598 (64·4%)	551 (63·0%)	1750 (64·3%)
	Mean (SD)	31·9 (15·8)	31·0 (15·7)	30·4 (15·8)	31·1 (15·7)
Sex					
	Female	456 (49·7%)	460 (49·5%)	438 (50·1%)	1354 (49·8%)
	Male	461 (50·3%)	469 (50·5%)	436 (49·9%)	1366 (50·2%)
Ethnic group					
	Mandinka	355 (38·7%)	470 (50·6%)	450 (51·5%)	1275 (46·9%)
	Jola	266 (29·0%)	251 (27·0%)	208 (23·8%)	725 (26·7%)
	Other	296 (32·3%)	208 (22·4%)	216 (24·7%)	720 (26·5%)
Maternal schooling					
	No school	449 (49·0%)	394 (42·4%)	404 (46·2%)	1247 (45·8%)
	1–9 years: lower or upper basic	316 (34·5%)	302 (32·5%)	297 (34·0%)	915 (33·6%)
	>9 years: secondary or college	152 (16·6%)	233 (25·1%)	173 (19·8%)	558 (20·5%)
Maternal occupation					
	At-home housewife	732 (79·8%)	664 (71·5%)	563 (64·4%)	1959 (72·0%)
	Small trader or non-skilled worker	115 (12·5%)	189 (20·3%)	248 (28·4%)	552 (20·3%)
	Professional and other	70 (7·6%)	76 (8·2%)	63 (7·2%)	209 (7·7%)
Height for age *Z* score <–2SD		204 (22·2%)	194 (20·9%)	147 (16·8%)	545 (20·0%)
Weight for height *Z* score <–2SD		60 (6·5%)	80 (8·6%)	56 (6·4%)	196 (7·2%)
Previous number of oral poliovirus vaccine doses, median (IQR)		6·0 (5·0–7·0)	6·0 (5·0–7·0)	6·0 (5·0–7·0)	6·0 (5·0–7·0)

Data presented as n (%) unless otherwise stated.

**Figure 2:**
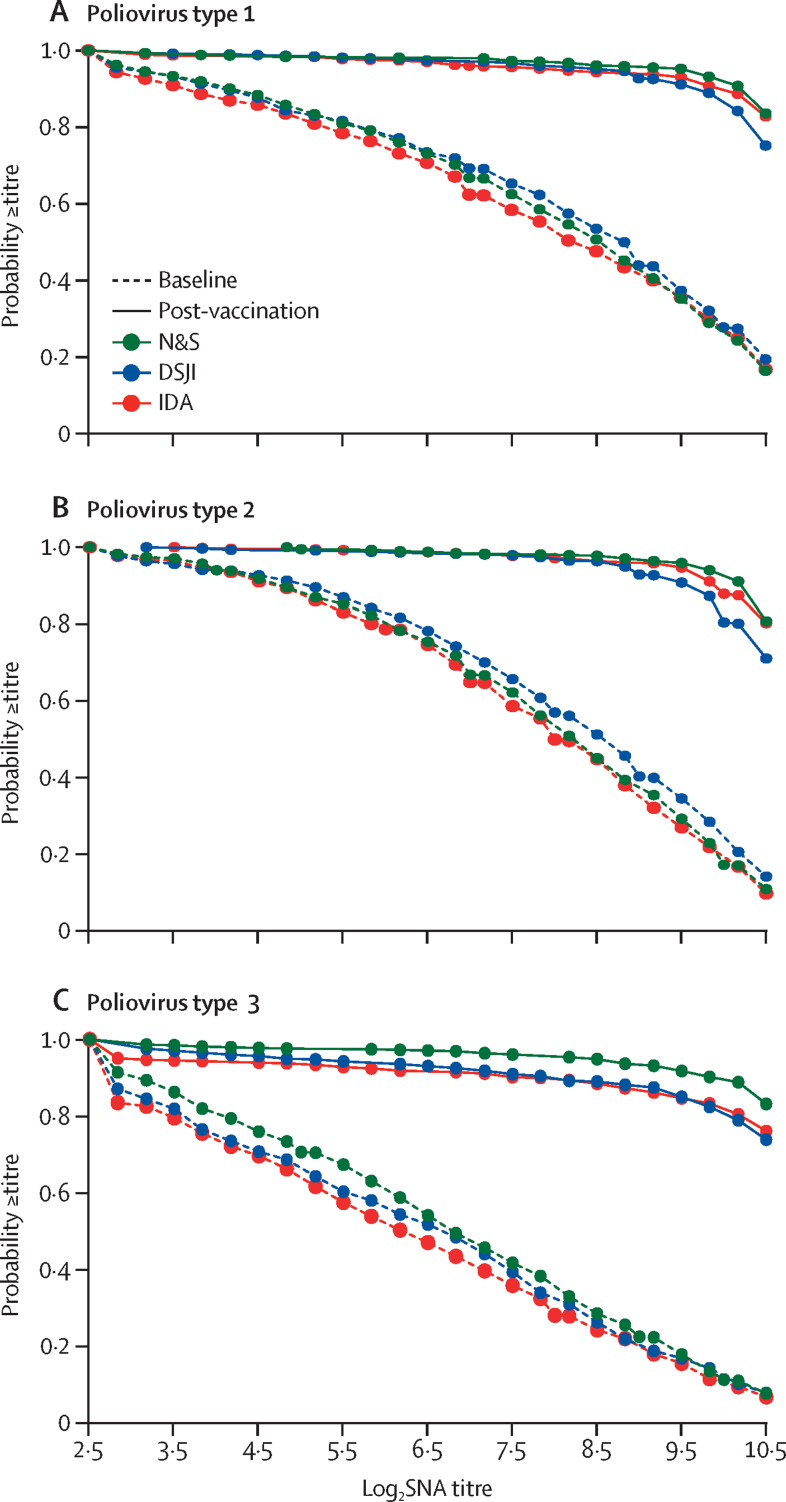
Distribution of SNA titres Reverse cumulative distribution curves illustrating the distribution of poliovirus type 1, poliovirus type 2, and poliovirus type 3 SNA titres at baseline and post-vaccination after the administration of an intradermal fractional dose of inactivated poliovirus vaccine using N&S, DSJI, or IDA. DSJI=disposable syringe jet injector. IDA=intradermal adapter. N&S=needle and syringe. SNA=serum neutralising antibody.

Children aged 24–59 months had received a median of seven (IQR 6–7) previous doses of trivalent OPV. The baseline seroprevalence in this group was 93·9% (95% CI 92·5–94·9; 1580/1683) for type 1, 96·9% (95·9–97·5; 1630/1683) for type 2, and 85·5% (83·8–87·1; 1440/1683) for type 3 (table 2). Baseline median antibody titres were 362 (274–362) for type 1, 274 (274–274) for type 2, and 91 (69–91) for type 3. There were no substantial differences in the distribution of baseline antibody titres across the three groups (figure 2).

**Table 2:** Baseline poliovirus serum neutralising antibody seroprevalence and median antibody titres in those aged 24–59 months

	**Needle and syringe (n=582)**	**Disposable syringe jet injector (n=574)**	**Intradermal adapter (n=527)**	**Total (n=1683)**
**Poliovirus type 1**				
Seroprevalence	550 (94·5%; 92·3–96·0)	542 (94·4%; 92·1–96·0)	488 (92·6%; 90·0–94·5)	1580 (93·9%; 92·5–94·9)
Median antibody titres	362 (274–362)	362 (362–446)	274 (223–362)	362 (274–362)
**Poliovirus type 2**				
Seroprevalence	567 (97·4%; 95·8–98·4)	553 (96·3%; 94·5–97·5)	510 (96·8%; 94·9–98·0)	1630 (96·9%; 95·9–97·5)
Median antibody titres	274 (223–274)	362 (274–362)	222 (223–274)	274 (274–274)
**Poliovirus type 3**				
Seroprevalence	520 (89·3%; 86·5–91·5)	485 (84·5%; 81·3–87·1)	435 (82·5%; 79·0–85·5)	1440 (85·6%; 83·8–87·1)
Median antibody titres	91 (91–111)	91 (69–111)	69 (56–91)	91 (69–91)

Data presented as n (%, 95% CI), or median (95% CI). Seroprevalence is defined as the number of participants with a serum neutralising antibody titre of ≥8 as a proportion of all participants tested.

The overall post-vaccination seroprevalence for poliovirus type 1 was 99·2% (95% CI 98·5–99·5; 1669/1683) and was consistent across administration methods (table 3). The immune response to type 1 ranged from 93·1% (95% CI 89·5–95·5; 268/288) in the DSJI group to 96·6% (94·0–98·0; 309/320) in the N&S group. All children who were seronegative for type 2 at baseline seroconverted, resulting in a 100% (99·8–100·0; 1683/1683) post-vaccination seroprevalence in all groups. Among those vaccinated using the DSJI, 90·1% (86·1–92·9; 281/312) had an immune response to type 2 compared with 93·8% (90·6–95·8; 331/353) of those vaccinated with N&S, and 96·6% (94·0–98·0; 316/327) of those vaccinated with IDA (table 3). The post-vaccination seroprevalence for type 3 ranged from 94·7% (92·4–96·3; 499/527) for the IDA group to 98·6% (97·3–99·3; 574/582) for the N&S group. Of the children who had fIPV administered using an N&S, 96·8% (94·5–98·0; 419/433) had an immune response to type 3, compared with 92·4% (89·5–94·5; 414/448) of those vaccinated with DSJI and 91·2% (88·0–93·5; 375/411) of those vaccinated with the IDA. There were no substantial differences in the distribution of antibody titres among those who received intradermal fIPV by each of the three administration methods (figure 2).

**Table 3:** Post-vaccination poliovirus SNA responses and median antibody titres in those aged 24–59 months

	**Needle and syringe (n=582)**	**Disposable syringe jet injector (n=574)**	**Intradermal adapter (n=527)**	**Total (n=1683)**
**Poliovirus type 1**				
Seroprevalence	578	570	521	1669
Seroprevalence %	99·3% (98·1–99·6)	99·3% (98·1–99·6)	98·9% (97·5–99·5)	99·2% (98·5–99·5)
Median antibody titres	≥1448 (1448–1448)	≥1448 (1448–1448)	≥1448 (1448–1448)	≥1448 (1448–1448)
Seroconversion	29/32	28/32	34/39	91/103
Seroconversion %	90·6% (75·8–96·8)	87·5% (71·9–95·0)	87·2% (73·3–94·4)	88·3% (80·6–93·1)
Four-fold titre rise	280/288	240/256	250/260	770/804
Four-fold titre rise %	97·2% (94·5–98·5)	93·8% (90·0–96·0)	96·2% (93·0–97·9)	95·8% (94·0–97·0)
Immune response	309/320	268/288	284/299	861/907
Immune response %	96·6% (94·0–98·0)	93·1% (89·5–95·5)	95·0% (91·9–96·9)	94·9% (93·3–96·1)
**Poliovirus type 2**				
Seroprevalence	582/582	574/574	527/527	1683/1683
Seroprevalence %	100·0% (99·3–100·0)	100·0% (99·3–100·0)	100·0% (99·3–100·0)	100·0% (99·8–100·0)
Median antibody titres	≥1448 (1448–1448)	≥1448 (1448–1448)	≥1448 (1448–1448)	≥1448 (1448–1448)
Seroconversion	15/15	21/21	17/17	53/53
Seroconversion %	100·0% (79·5–100·0)	100·0% (84·5–100·0)	100·0% (81·5–100·0)	100·0% (93·1–100·0)
Four-fold titre rise	316/338	260/291	299/310	875/939
Four-fold titre rise %	93·5% (90·3–95·6)	89·3% (85·3–92·4)	96·5% (93·8–98·0)	93·2% (91·4–94·5)
Immune response	331/353	281/312	316/327	928/992
Immune response %	93·8% (90·6–95·8)	90·1% (86·1–92·9)	96·6% (94·0–98·0)	93·5% (91·8–94·9)
**Poliovirus type 3**				
Seroprevalence	574/582	560/574	499/527	1633/1683
Seroprevalence %	98·6% (97·3–99·3)	97·6% (95·9–98·5)	94·7% (92·4–96·3)	97·0% (96·0–97·6)
Median antibody titres	≥1448 (1448–1448)	≥1448 (1448–1448)	≥1448 (1448–1448)	≥1448 (1448–1448)
Seroconversion	54/62	75/89	70/92	199/243
Seroconversion %	87·1% (76·5–93·3)	84·3% (75·3–90·4)	76·1% (66·4–83·5)	81·9% (76·5–86·1)
Four-fold titre rise	365/371	339/359	305/319	1009/1049
Four-fold titre rise %	98·4% (96·5–99·3)	94·4% (91·5–96·4)	95·6% (92·8–97·4)	96·2% (94·8–97·1)
Immune response	419/433	414/448	375/411	1208/1292
Immune response %	96·8% (94·5–98·0)	92·4% (89·5–94·5)	91·2% (88·0–93·5)	93·5% (92·0–94·6)

Data presented as n/N, % (95% CI), or median (95% CI). Seroprevalence is defined as the number of participants with an SNA titre of ≥8 as a proportion of all participants tested. The percentage of children who had an immune response after intradermal fractional dose inactivated poliovirus vaccine was calculated combining the percentage who underwent seroconversion (baseline SNA titres of <8 and a post-vaccination titre of ≥8) with the percentage who were seropositive (SNA ≥8) at baseline and had a four-fold rise in SNA titres post-vaccination. Children with a baseline titre of >362 were excluded from the analysis as a four-fold rise was beyond the upper limit of quantification the assay. SNA=serum neutralising antibody.

**Figure 3:**
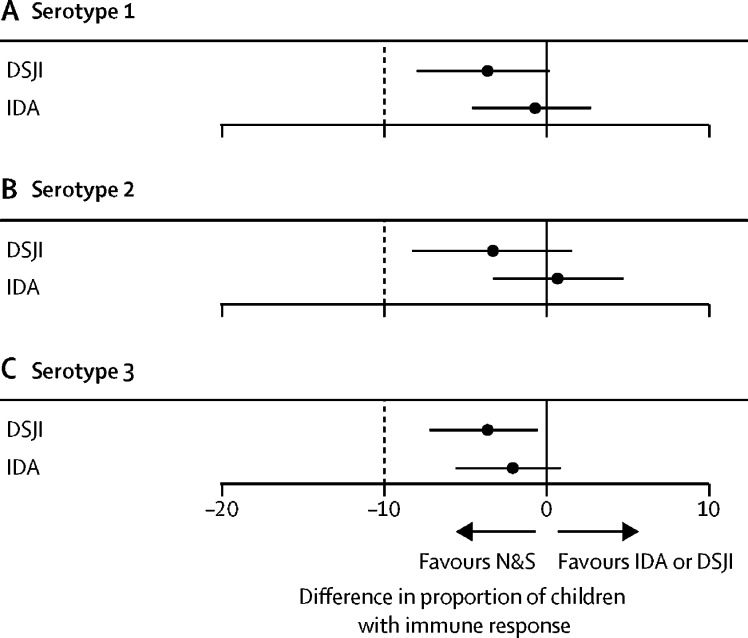
Effects of administration method on type-specific immune responses Differences in the percentage of participants having an immune response to poliovirus type 1, poliovirus type 2, or poliovirus type 3 after the administration of an intradermal fractional dose of inactivated poliovirus vaccine via IDA or DSJI, compared with the reference N&S method. The percentage of children who had an immune response after intradermal fractional dose inactivated poliovirus vaccine was calculated combining the percentage who underwent seroconversion (baseline SNA titres of <8 and a post-vaccination titre of ≥8) with the percentage who were seropositive (SNA≥8) at baseline and had a four-fold rise in SNA titres post-vaccination. Children with a baseline titre of >362 were excluded from the analysis because a four-fold rise was beyond the upper limit of quantification the assay (table 3). Point estimates and 97·5% CI are illustrated. The 97·5% CI were adjusted for age, sex, and variables associated with the immune response in a multivariable regression model developed for each type (type 1: baseline seropositivity, appendix p 8; type 2: number of previous oral poliovirus vaccine doses, time taken to vaccinate, maternal occupation, appendix p 9; type 3: baseline seropositivity, appendix p 10). DSJI=disposable syringe jet injector. IDA=intradermal adapter. N&S=needle and syringe. SNA=serum neutralising antibody.

For the primary non-inferiority analysis, the type 2 immune response rates in those who received fIPV using either the DSJI or the IDA were non-inferior to the immune response rates in those who had the vaccine administered with an N&S (figure 3). The adjusted difference was −3·3% (97·5% CI −8·3 to 1·5) for DSJI, and 0·7% (−3·3 to 4·7) for IDA administration. The unadjusted differences were −3·7% (−8·7 to 1·1) for DSJI and 2·9% (−0·9 to 6·8) for IDA. The adjusted difference in the immune response to types 1 and 3 after administration by either of the alternative administration methods were also non-inferior to the immune response rates to the same types generated after N&S administration. The inferences from the non-inferiority tests were unchanged by adjustment for baseline variables (appendix p 7).

Baseline seropositivity was associated with an increase in immune response rates for types 1 and 3. There were no other consistent associations with the other variables analysed (appendix pp 8–10). Neither the designation of the vaccinator (public health officers compared with nurses, odds ratio [OR] 0·70 [95% CI 0·40–1·23]), nor the time taken to administer the vaccine (1–<2 mins *vs* <1 min, OR 1·05 [95% CI 0·67–1·64]; ≥2 mins *vs* <1 min, OR 0·75 [95% CI 0·20–2·82]) affected on the immune response rates (appendix p 11). The amount of clustering at a team level was low (intraclass correlation coefficients: type 1, 0·000; type 2, 0·003; and type 3, 0·012) for the individual serotype analyses. For the combined serotype analyses, the team-level clustering was again low (intraclass correlation coefficient 0·030), whereas, as expected, there was considerable clustering in the response to the three poliovirus types within individuals (intraclass correlation coefficient 0·520).

The median diameter of the intradermal fluid bleb generated by the DSJI was 3 mm (IQR 1–5) compared with 5 mm (4–7) with the IDA and 7 mm (5–8) with the N&S. A fifth of children (172/864; 20·0%) lost at least 20 μL of fluid onto the skin at the time of the injection with the DSJI compared with only approximately 1% of children who lost this volume with N&S (12/880; 1·4%) or IDA (8/849; 0·9%; table 4). There was no independent association between the size of the intradermal fluid bleb and immune response once administration method had been accounted for (appendix p 11). There was no association between the amount of fluid lost onto the skin and immune response. There was a weak association between overall injection quality and immune response for type 2 (OR 1·95; 95% CI 1·00–3·79) but no association for the other types on adjusted analysis (appendix pp 12–13).

**Table 4:** Vaccine delivery outcomes

		**Needle and syringe (n=917)**	**Disposable syringe jet injector (n=929)**	**Intradermal adapter (n=874)**
Intradermal fluid bleb size, mm		7 (5–8)	3 (1–5)	5 (4–7)
Fluid loss onto skin*				
	Dry	317/880 (36·0%)	288/864 (33·3%)	546/849 (64·3%)
	<5 μL	505/880 (57·4%)	263/864 (30·4%)	258/849 (30·4%)
	5 to <10 μL	25/880 (2·8%)	76/864 (8·8%)	26/849 (3·1%)
	10 to <20 μL	21/880 (2·4%)	65/864 (7·5%)	11/849 (1·3%)
	≥20 μL	12/880 (1·4%)	172/864 (19·9%)	8/849 (0·9%)
Time taken to vaccinate				
	<1 min	300/917 (32·7%)	507/929 (54·6%)	294/874 (33·6%)
	1 to <2 min	592/917 (64·6%)	409/929 (44·0%)	553/874 (63·3%)
	2 to <3 min	23/917 (2·5%)	11/929 (1·2%)	24/874 (2·7%)
	≥3 min	2/917 (0·2%)	2/929 (0·2%)	3/874 (0·3%)
Distress associated with injection				
	Did not cry	427/917 (46·6%)	771/929 (83·0%)	392/874 (44·9%)
	Cried briefly, consoled easily	329/917 (35·9%)	66/929 (7·1%)	351/874 (40·2%)
	Crying for a long time	35/917 (3·8%)	12/929 (1·3%)	23/874 (2·6%)
	Crying before injection	126/917 (13·7%)	80/929 (8·6%)	108/874 (12·4%)

Data presented as median (IQR) or n/N (%).

* Fluid loss was not collected on every participant because of the rate of vaccination.

More than half of injections administered by DSJI (507/929; 54·6%) were completed in under 1 min compared with approximately a third of injections administered by N&S (300/917; 32·7%) and IDA (294/874; 33·6%; table 4). More than 95% of injections were completed in under 2 min. More than 80% of children (771/929; 83·0%) did not cry when the intradermal fIPV was administered using the DSJI compared with approximately 45% of children after N&S-based (427/917; 46·6%) or IDA-based (392/874; 44·9%) administration. More children were crying before receiving an injection with N&S or IDA methods. A mean of 63 (SD 5·0) fIPV doses were obtained from each 10-dose vial using the DSJI compared with a mean of 50 (SD 2·7) doses using the N&S.

Vaccinations were well tolerated. A low number of solicited injection-site adverse events occurred irrespective of administration method (appendix pp 14–16). On day 3, five (0·2%) of 2701 patients had any tenderness, one (<0·1%) patient had any erythema, and seven (0·3%) had any induration, and all reactions were resolved without intervention. Overall, 99·4% (2684/2701) of children had an axillary temperature of less than 37·5°C. No child had a temperature of more than 39·0°C, and the small number of children with a low-grade fever required treatment with only simple antipyretics. The rates of solicited systemic adverse events in children were also low. Most complaints were mild or moderate in severity and all resolved with no more than symptomatic treatment. There were no notable differences in the rates of solicited systemic adverse events between the three administration methods. A total of 728 unsolicited adverse events were recorded during the 4-week follow-up period (appendix p 17): 234 in the N&S-based administration group, 219 in the DSJI-based administration group, and 275 in the IDA-based administration group. Upper respiratory tract infections (196/728; 26·9%) and gastroenteritis or diarrhoea (193/728; 26·5%) were the most common adverse events. Overall, 181 of 917 participants (19·7%) in the N&S group, 178 of 929 participants (19·2%) in the DSJI group, and 211 of 874 participants (24·1%) in the IDA group had at least one unsolicited adverse event. Six children, two who received fIPV by each of the three administration methods, had a serious adverse event during the study. Four children were admitted to hospital with a diagnosis of gastroenteritis or diarrhoea, one with a skin infection, and one with contusions after a road traffic accident. None of the serious adverse events were related to vaccination and all made a full recovery without sequelae.

## Discussion

This trial established the immunogenicity and safety of intradermal fIPV when administered with an N&S, a DSJI, and an IDA in The Gambia. These results should allay concerns regarding the feasibility of delivering intradermal injections in large-scale outbreak response campaigns. For all three poliovirus types, the immunogenicity of intradermal fIPV administered with either the DSJI or with the IDA was non-inferior to the immunogenicity of the same vaccine administered with an N&S. The vaccination was well tolerated by all three administration methods.

The immunogenicity of intradermal fIPV has been examined in previous randomised controlled trials, albeit only one in sub-Saharan Africa.^10^, ^15^, ^16^, ^17^, ^18^, ^26^, ^28^, ^29^ Two meta-analyses published in 2019 and 2021 compared equivalent full-dose and fIPV dose schedules. Type 2 seroconversion rates after one and in some cases two and three fIPV doses are lower than after the equivalent full-dose schedules and median antibody titres tend to be lower, irrespective of the dose number.^18^, ^30^ Nonetheless, fIPV used to boost immunity after OPV in infants results in substantially higher type 2 immune response rates than further OPV doses.^28^ In trials conducted in low-income and middle-income countries, type 2 seroprevalence in young children boosted with intradermal fIPV after OPV ranges from approximately 90% to close to 100%.^16^, ^26^, ^28^, ^31^ Two fractional doses of IPV are more immunogenic than a single full dose at the same time as being dose sparing.^15^, ^29^ Fractional as well as full-dose IPV has also been shown to boost mucosal immunity in those who have previously received the oral vaccine, making it suitable for outbreak control.^10^, ^17^

Despite such supportive data, a persistent concern regarding the rapid deployment of intradermal fIPV in campaigns to control cVDPV2 outbreaks is the feasibility of delivering intradermal injections on a large scale. Hence, there are concerns that the immunogenicity and safety data generated in trials might not be replicated during an outbreak. In 2016, more than 300 000 children were vaccinated with intradermal fIPV using N&S within 14 days of detection of VDPV2 in Telangana, India. Although successful in terms of coverage, a large number of trained vaccinators had to be brought into the target area from other districts, which will not be feasible in many settings.^32^ A report after another intradermal fIPV campaign, undertaken in Hyderabad, Pakistan, recorded little experience and incorrect vaccine administration as limitations to the use of N&S.^33^ A study conducted in Karachi, Pakistan, during a catch-up campaign supported the feasibility of DSJI use in this context and also reported that 97·6% (578/592) of vaccinators and 99·6% (4792/4813) of caregivers reported a preference for DSJI over the previous experience of intramuscular N&S vaccination.^34^ Immunogenicity data were not collected in these campaigns and safety data were limited to the routine passive reporting of adverse events after immunisation.

This study provides compelling data to support the use of intradermal fIPV in cVDPV2 outbreak response campaigns, particularly in sub-Saharan Africa where they are most needed based on a rapidly increasing number of cVDPV2 outbreaks being reported. Neither the safety nor immunogenicity of intradermal fIPV was affected by the administration method used. All children who were seronegative to poliovirus type 2 at baseline seroconverted in response to a single dose of intradermal fIPV. High SNA titres were generated against all three poliovirus types, irrespective of the administration method. There was a weak association between injection quality and the immune response for poliovirus type 2. However, the titres generated by all three administration methods were consistently high despite the difference in markers of injection quality. Because 20% of injections with the DSJI were associated with more than 20% vaccine loss, further dose reductions might be possible. The little effect that the previous experience administering intradermal injections of the vaccinator had on immune response rates also suggests that this is not a crucial determinant. The perceived importance of injection quality, reflecting the delivery of fIPV into the dermis rather than deeper tissue, is based on the presence of distinct antigen-presenting cells in this layer of the skin, considered able to compensate for the reduced antigen dose.^35^ Although some adult data support this notion, a trial in infants suggests that fIPV delivered intramuscularly might offer similar protection to doses administered by the intradermal route.^36^, ^37^ Our study also suggests that the immune responses generated by fIPV are robust and that the route of administration should not be a barrier, even when there are few experienced personnel.

More than 25% more doses were obtained from each ten-dose vial when using the DSJI method than when using the N&S method. This increase reflects the standard requirement to overfill vials to account for the dead space in an N&S. Dead space in the DSJI has been minimised, with any overage drawn out of the vial during filling being returned to the vial as part of the priming process. In an outbreak campaign, the scale and focus of which are necessarily established by IPV availability, this provides the potential to increase campaign coverage by stretching the vaccine supply.^2^

Both the DSJI and the IDA have been endorsed by the Strategy Committee of the Global Polio Eradication Initiative for use in polio outbreak responses. The DSJI has been prequalified by WHO for intradermal immunisation. Although the unit cost of both delivery methods is considerably higher than for N&S delivery, analysis suggests that this cost might be largely offset when the effect of dose sparing and of operational costs, including the training of health workers, is included.^38^

The pragmatic nature of the trial, aiming to align procedures with those used to deliver campaigns with parenteral vaccines in The Gambia and other countries in sub-Saharan Africa, was a key strength of the study.^19^ These are the first immunogenicity and systematically collected safety data of intradermal fIPV delivered in the context of a campaign. Indeed, to the best of our knowledge, the trial design is novel and could be applied to other vaccines used in outbreak control or delivered through campaigns. Few children were excluded based on ineligibility. Although approximately a fifth of children were at least moderately stunted in height, this did not affect immune response rates, which should reassure those planning campaigns in other settings with high rates of malnutrition. The sample size was also exceeded, adding to the strength of the safety data and the confidence that can be placed in the immunogenicity data.

The trial had some limitations. Participants were not individually randomly assigned, but rather had the vaccine administered using the method being used at the site they attended. Similarly, the parents were not masked. Although inherent to the trial design, both result in a risk of selection bias. Although there were some differences in the baseline demographic characteristics between groups, adjusting for these variables did not alter the inference from the non-inferiority analysis, and any effect is likely to be small given that the laboratory assessment was blinded. The number of vaccination teams was kept intentionally low to ensure a continuous flow of vaccinees at each vaccination point during the campaigns. We ensured the previous vaccination experience within each of the two vaccinator groups was similar. Nonetheless, each vaccinator's inherent proficiency might have been different. The immunogenicity assessment was undertaken in those between 24 and 59 months of age who had almost universally received previous trivalent OPV but who had not received IPV. In cVDPV2 outbreak campaigns, intradermal fIPV will be used in conjunction with a type 2 OPV. Thus, although increasingly few children will have been primed with trivalent OPV, intradermal fIPV will still generally be given after at least one dose of a type 2 OPV. In addition, children born since approximately 2015 might, based on WHO recommendations, have been primed with IPV, which is likely to enhance the humoral responses to the intradermal fIPV boost.

A novel, more genetically stable type 2 OPV has been granted interim WHO Emergency Use Listing, allowing limited use in 2021 and, subsequently, after WHO prequalification, on a more widespread basis.^2^, ^39^ Reversion to virulence with novel OPV2 is expected to be less frequent than with monovalent OPV type 2. However, given the ongoing circulation of cVDPV2, West Africa and other parts of sub-Saharan Africa, the need to optimally deploy IPV to prevent avoidable paralytic disease remains high. This need has only been increased by the SARS-CoV-2 pandemic given that campaigns have been halted and surveillance compromised.^40^ This trial provides strong data to support the use of intradermal fIPV in campaigns in sub-Saharan Africa and other similar settings, and should assuage any persistent concerns that might limit the future use of this crucial public health intervention.

## Data sharing

The individual participant data that underlie the results reported in this Article, after de-identification (in the text, tables, figures, and appendices), will be shared. Individual participant data will be available from the time the manuscript is published. Supporting clinical documents including the study protocol and the informed consent form will be available. Researchers who provide a scientifically sound proposal will be allowed access to the individual participant data. Proposals should be directed to the corresponding author. These proposals will be reviewed and approved by a panel of senior scientific personnel from the Medical Research Council Unit in The Gambia at London School of Hygiene & Tropical Medicine and by the Gambian Government and Medical Research Council Joint Ethics Committee. To gain access, data requesters will need to sign a data access agreement.

## Declaration of interests

We declare no competing interests.
