# Supplementary material for: Intradermal administration of fractional doses of the inactivated poliovirus vaccine in a campaign: a pragmatic, open-label, non-inferiority trial in The Gambia
Source: Lancet Glob Health. 2021 Dec 21;10(2):e257–68. doi: 10.1016/S2214-109X(21)00497-6 (PMC8786671; doi:10.1016/S2214-109X(21)00497-6)
Supplement: Supplementary appendix [file mmc1.pdf]

THE LANCET
Global Health

## Supplementary appendix

This appendix formed part of the original submission and has been peer reviewed. We post it as supplied by the authors.

Supplement to: Bashorun AO, Badjie Hydara M, Adigweme I, et al. Intradermal administration of fractional doses of the inactivated poliovirus vaccine in a campaign: a pragmatic, open-label, non-inferiority trial in The Gambia. *Lancet Glob Health* 2021; published online Dec 21. https://doi.org/10.1016/S2214-109X(21)00497-6.

## Supplementary Tables

**Figure S1: Overview of trial schedule.**

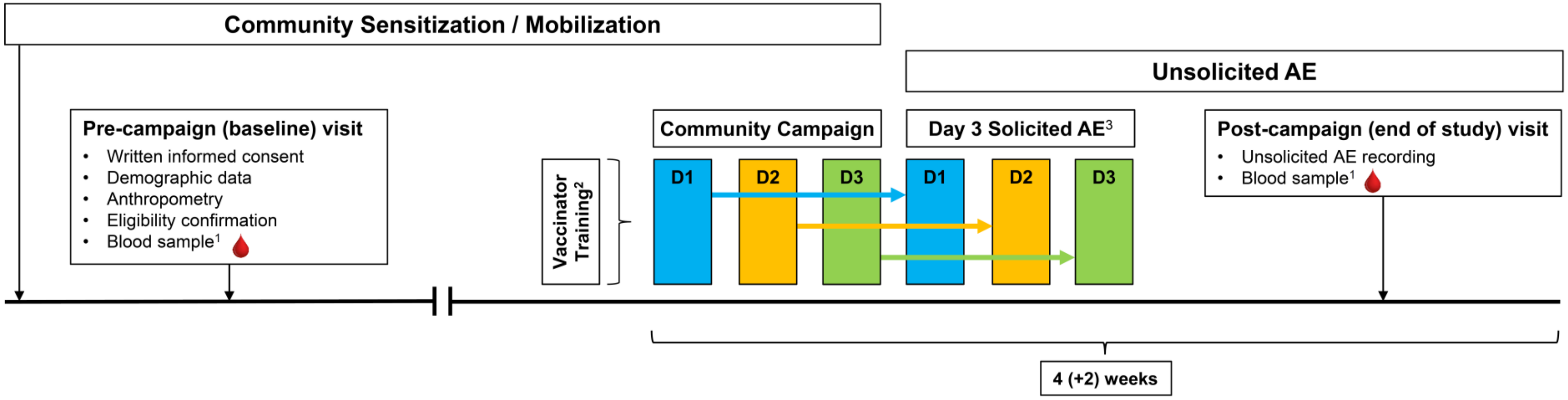


[1] 2.0 mL 24-59-month-olds only; [2] Up to 2 hours on each administration method; [3] Tenderness, erythema, induration, fever, diarrhoea, vomiting, reduced feeding/eating, drowsiness, irritability (Table S1); AE – adverse events; D – day.

**Figure S2: Process for allocation of given vaccination teams to given administration methods.** Each team consisted of a public health officer experienced at giving ID injections and a nurse with little or no ID injection experience. N&S – needle and syringe; DSJI – disposable syringe jet injector; IDA – intradermal device

| | Team | Day 1 | Day 2 | Day 3 | Total vaccinees per team |
|---|---|---|---|---|---|
| Six x 2 person vaccination teams defined and trained ↓ Allocated to cover a given geographical area on each day of campaign ↓ Randomised (A – F) | A | N&S | DSJI | IDA | ~ 300 |
| | B | IDA | N&S | DSJI | ~ 300 |
| | C | DSJI | IDA | N&S | ~ 300 |
| | D | N&S | N&S | N&S | ~ 300 |
| | E | DSJI | DSJI | DSJI | ~ 300 |
| | F | IDA | IDA | IDA | ~ 300 |
| | Total vaccinees per day | ~ 600 | ~ 600 | ~600 | **~ 1800** |

**Figure S3: (A) Auto-disable 0.1 mL Needle and Syringe; (B) Disposable Syringe Jet Injector (Tropis®); (C) Intradermal Adaptor.**

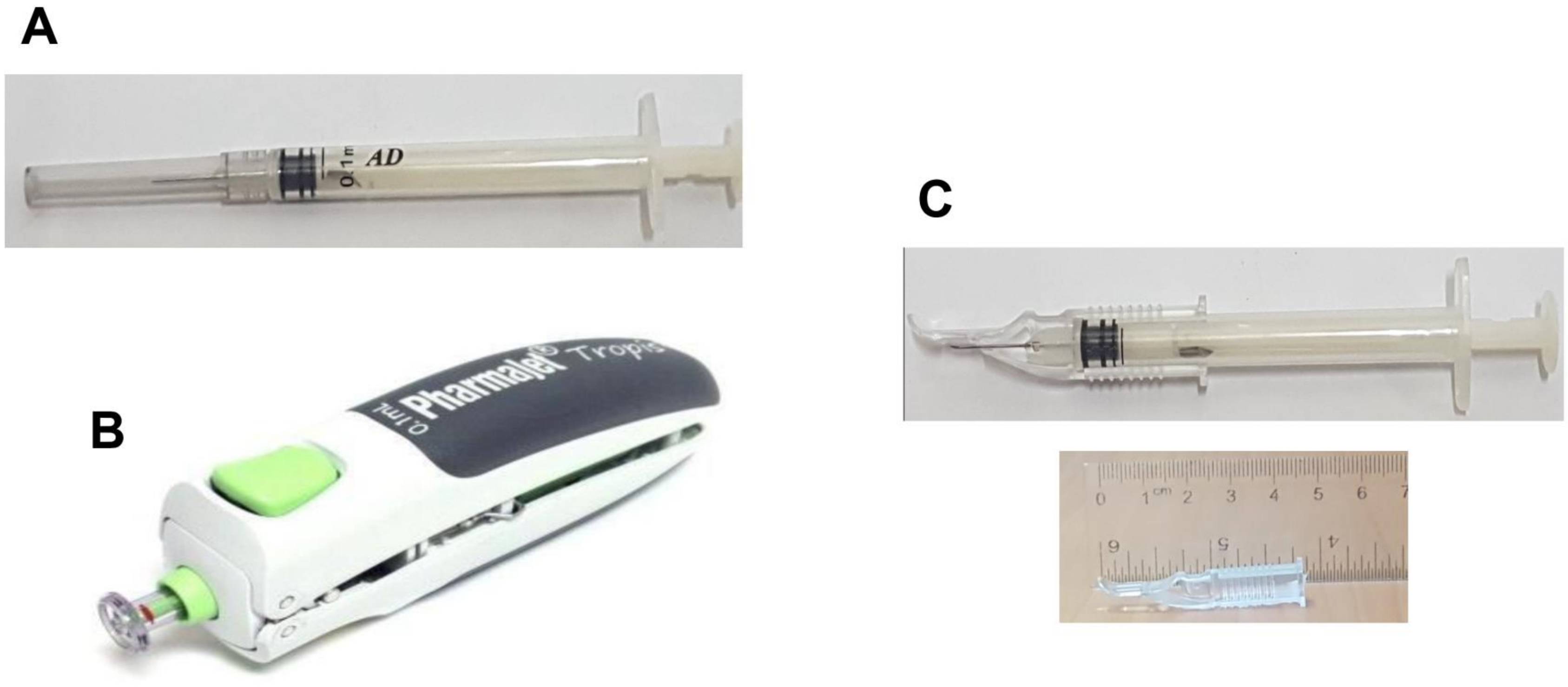

## Supplementary Tables

**Table S1: Temporal changes in polio vaccination in The Gambia impacting on study participants prior polio vaccination history.**

| | October 2013 | Pre-April 2015 EPI schedules | April 2015 to April 2016 EPI schedule | Post April 2016 |
|---|---|---|---|---|
| **Birth** | | tOPV | tOPV | bOPV |
| **2 months** | tOPV campaign targeting 6-week-olds to <5-year-olds | tOPV | tOPV | bOPV |
| **3 months** | | tOPV | tOPV | bOPV |
| **4 months** | | tOPV | tOPV plus IPV | bOPV plus IPV |
| **9 months** | | tOPV | tOPV | bOPV |
| **18 months** | | tOPV | tOPV | bOPV |

tOPV – trivalent oral poliovirus vaccine; IPV – inactivated poliovirus vaccine; bOPV – bivalent poliovirus vaccine

## Supplementary Tables

# Table S2: Injection site and systemic adverse event grading criteria

| Local | Grade 0 (normal) | Grade 1 | Grade 2 | Grade 3 | Grade 4 |
|---|---|---|---|---|---|
| Tenderness | No tenderness to touch | Mild discomfort to touch only. | Cries or obviously distressed by touch | Cries or obviously distressed by passive limb movement | Requires hospitalization for the management of tenderness |
| Erythema/redness (size as well as grade will be collected) | No erythema | Erythema present but ≤ 2.5cm in diameter | Erythema present and >2.5cm in diameter but with <50% of the surface area of the extremity segment (e.g. thigh) involved | Erythema affecting ≥ 50% of the surface area of the extremity segment (e.g. thigh) involved OR ulceration OR secondary infection OR phlebitis OR sterile abscess OR drainage | Potentially life-threatening consequences (e.g. Necrosis involving dermis or deeper tissues or exfoliative dermatitis) requiring intervention |
| Induration/swelling (size as well as grade will be collected) | No induration | Induration present but ≤ 2.5cm in diameter | Induration present and >2.5cm in diameter but with <50% of the surface area of the extremity segment (e.g. thigh) involved | Induration affecting ≥ 50% of the surface area of the extremity segment (e.g. thigh) involved OR ulceration OR secondary infection OR phlebitis OR sterile abscess OR drainage | Potentially life-threatening consequences (e.g. Necrosis involving dermis or deeper tissues or exfoliative dermatitis) requiring intervention |
| **Systemic** | | | | | |
| Acute systemic allergic reactions | No acute reaction | Local urticaria | Mild angiodema | Symptomatic bronchospasm (wheeze) | Anaphylaxis, life threatening bronchospasm (wheeze) or laryngeal oedema (stridor) |
| Temperature | <37.5˚C | 37.5 - 37.9˚C | 38.0 - 38.9˚C | 39.0 - 39.9˚C | ≥ 40˚C |
| Vomiting | No vomiting | 1-2 episodes in 24 hours | >2 episodes/24 hours | Requires naso-gastric rehydration | Results in hypotension or requires iv hydration and inpatient hospital treatment |
| Diarrhoea | Normal stool consistency and frequency | 2 - 3 loose stools in 24 hours | 4-5 loose stools in 24 hours | 6 or more loose stools in 24 hours | Results in hypotension or requires iv hydration and inpatient hospital treatment |
| Reduced feeding/eating | Feeding/eating normally | Feeding/eating minimally reduced compared to normal | Feeding/eating significantly reduced compared to normal | Not feeding/eating at all | Requires hospitalization due to lack of feeding/eating |
| Drowsiness | Normal level of alertness/ behaviour | Sleeping more than normal but wakes easily. Not interfering with activities for age (e.g. feeding/play) | Sleeping more than normal. Somewhat difficult to wake, some interference with normal activities for age (e.g. feeding/play) | Very sleepy, difficult to wake and wakes only briefly. Prevents normal activities for age (e.g. feeding/play) | Requires hospitalization due to drowsiness |
| Irritability | Normal behaviour. Not irritable | Crying more than normal but settles/ comforted easily | Crying more than normal, difficult to settle/comfort and affecting normal activities for age (e.g. feeding/play) | Crying more than normal and cannot be settled or comforted. Preventing normal activities (e.g. feeding/play) | Requires hospitalization due to irritability |

Derived from the Division of AIDS Table for Grading the Severity of Adult and Pediatric Adverse Events (Version 2.0, November 2014) and the Common Terminology Criteria for Adverse Events (CTCAE) Version 4.0, Published May 28, 2009 (v4.03, June 14, 2010)

**Table S3: Baseline demographic and anthropometric characteristics (≥ 24 months of age)**

| | N&S (N=601) | DSJI (N=598) | IDA (N=551) | Total (N=1750) |
|---|---|---|---|---|
| **Age (months)** Mean (SD) | 41.4 (9.9) | 40.6 (10.3) | 40.5 (10.0) | 40.8 (10.1) |
| **Sex** Male n (%) | 312 (51.9%) | 294 (49.2%) | 269 (48.8%) | 875 (50.0%) |
| **Ethnic Group** | | | | |
| Mandinka n (%) | 228 (37.9%) | 301 (50.3%) | 281 (51.0%) | 810 (46.3%) |
| Jola n (%) | 168 (28.0%) | 154 (25.8%) | 136 (24.7%) | 458 (26.2%) |
| Other n (%) | 205 (34.1%) | 143 (23.9%) | 134 (24.3%) | 482 (27.5%) |
| **Maternal schooling** | | | | |
| No school n (%) | 308 (51.2%) | 266 (44.5%) | 258 (46.8%) | 832 (47.5%) |
| 1-9 years: Lower/Upper Basic n (%) | 201 (33.4%) | 183 (30.6%) | 195 (35.4%) | 579 (33.1%) |
| >9 years: Secondary/College n (%) | 92 (15.3%) | 149 (24.9%) | 98 (17.8%) | 339 (19.4%) |
| **Maternal occupation** | | | | |
| At home, housewife n (%) | 471 (78.4%) | 418 (69.9%) | 350 (63.5%) | 1239 (70.8%) |
| Small trader, non-skilled worker n (%) | 83 (13.8%) | 127 (21.2%) | 163 (29.6%) | 373 (21.3%) |
| Professional and other n (%) | 47 (7.8%) | 53 (8.9%) | 38 (6.9%) | 138 (7.9%) |
| **Height for age z-score** < -2SD n (%) | 140 (23.3%) | 122 (20.4%) | 88 (16.0%) | 350 (20.0%) |
| **Weight for height z-score** < -2SD n (%) | 38 (6.3%) | 35 (5.9%) | 31 (5.6%) | 104 (5.9%) |
| **Prior number of OPV doses** (Median (IQR)) | 7.0 (6.0 - 8.0) | 7.0 (6.0 - 7.0) | 7.0 (6.0 - 7.0) | 7.0 (6.0 - 7.0) |

N&S – needle and syringe; DSJI – disposable syringe jet injector; IDA – intradermal device; IQR – interquartile range; SD - standard deviation

**Table S4: Unadjusted and adjusted differences in the immune response rates comparing DSJI or IDA based fIPV administration with N&S based administration.**

| **Serotype 2** | | **$IR_{DSJI/IDA}$ – $IR_{N\&S}$ (%, [97.5% CI])** |
|---|---|---|
| DSJI | Unadjusted | -3.7 (-8.7 to 1.1) |
| | Adjusted§ | -3.3 (-8.3 to 1.5) |
| IDA | Unadjusted | 2.9 (-0.9 to 6.8) |
| | Adjusted§ | 0.7 (-3.3 to 4.7) |
| **Serotype 1** | | |
| DSJI | Unadjusted | -3.5 (-8.0 to 0.6) |
| | Adjusted‡ | -3.7 (-8.0 to 0.1) |
| IDA | Unadjusted | -1.6 (-5.6 to 2.2) |
| | Adjusted‡ | -0.8 (-4.6 to 2.7) |
| **Serotype 3** | | |
| DSJI | Unadjusted | -4.4 (-8.0 to -0.9) |
| | Adjusted‖ | -3.7 (-7.2 to -0.6) |
| IDA | Unadjusted | -5.5 (-9.4 to -1.9) |
| | Adjusted‖ | -2.1 (-5.6 to 0.8) |

DSJI – disposable syringe jet injector; IDA – intradermal device; CI – confidence intervals; adjusted for § age, sex, baseline seropositivity; ‡ age, sex, number of prior OPV doses, time taken to vaccinate, maternal occupation; ‖ age, sex, baseline seropositivity

**Table S5: Multilevel logistic regression model examining the associations with serotype 1 immune response rates allowing for clustering within vaccination teams.**

| Serotype 1 | | | Unadjusted | | Adjusted | |
|---|---|---|---|---|---|---|
| | | N | OR (95% CI) | p-value | OR (95% CI) | p-value |
| Administration method | N&S | 320 | | | | |
| | IDA | 299 | 0.67 (0.30-1.49) | 0.33 | 0.71 (0.32-1.59) | 0.412 |
| | DSJI | 288 | 0.48 (0.22-1.01) | 0.054 | 0.48 (0.22-1.03) | 0.058 |
| Age (months) | 24 - <36 | 270 | | | | |
| | 36 - <48 | 316 | 1.34 (0.67-2.68) | 0.409 | 1.38 (0.68-2.79) | 0.369 |
| | ≥ 48 | 321 | 1.84 (0.87-3.89) | 0.111 | 1.85 (0.87-3.94) | 0.111 |
| Sex | Male | 455 | | | | |
| | Female | 452 | 0.99 (0.55-1.80) | 0.982 | 1.05 (0.57-1.91) | 0.880 |
| Ethnicity | Mandinka | 406 | | | | |
| | Jola | 247 | 0.67 (0.32-1.39) | 0.28 | | |
| | Other | 254 | 0.74 (0.35-1.56) | 0.424 | | |
| Height-for-age z score | > -2 | 712 | | | | |
| | ≤ -2 | 195 | 0.99 (0.48-2.02) | 0.968 | | |
| Prior number of OPV doses | 0-5 | 108 | | | | |
| | 6-7 | 600 | 1.93 (0.88-4.23) | 0.1 | | |
| | 8-10 | 199 | 1.72 (0.68-4.37) | 0.255 | | |
| Designation of vaccinator | Nurse | 529 | | | | |
| | PHO | 378 | 0.93 (0.51-1.68) | 0.799 | | |
| Time taken to vaccinate (minutes) | < 1 | 393 | | | | |
| | 1 - <2 | 494 | 1.33 (0.73-2.43) | 0.347 | | |
| | ≥ 2 | 20 | 1.18 (0.15-9.22) | 0.874 | | |
| Maternal education§ | No school | 430 | | | | |
| | 1-9 years | 314 | 1.00 (0.52-1.95) | 0.99 | | |
| | >9 years | 163 | 1.04 (0.46-2.40) | 0.918 | | |
| Maternal occupation‡ | At home | 650 | | | | |
| | Small trade | 188 | 0.68 (0.34-1.37) | 0.284 | | |
| | Profession | 69 | 0.60 (0.22-1.60) | 0.305 | | |
| Baseline seropositivity | Negative | 103 | | | | |
| | Positive | 804 | 2.99 (1.49-5.97) | 0.002 | 2.99 (1.49- 6.03) | 0.002 |
| ICC | Team: 0 | | | | | |

N&S – needle and syringe; IDA – intradermal adapter; DSJI – disposable syringe jet injector; PHO – public health officer; OPV – oral poliovirus vaccine; OR – odds ratio; CI – confidence interval; ICC – Intraclass correlation coefficient; § 1-9 years: lower and upper basic school, >9 years: secondary and/or college education; ‡ small trade: small trader/unskilled worker, profession: professional and other skilled work

**Table S6: Multilevel logistic regression model examining the associations with serotype 2 immune response rates allowing for clustering within vaccination teams.**

| Serotype 2 | | | Unadjusted | | Adjusted | |
|---|---|---|---|---|---|---|
| | | N | OR (95% CI) | p-value | OR (95% CI) | p-value |
| Administration method | N&S | 353 | | | | |
| | IDA | 327 | 1.94 (0.88-4.25) | 0.098 | 1.62 (0.75-3.52) | 0.222 |
| | DSJI | 312 | 0.55 (0.28-1.09) | 0.087 | 0.48 (0.24-0.97) | 0.041 |
| Age (months) | 24 - <36 | 279 | | | | |
| | 36 - <48 | 344 | 0.66 (0.35-1.25) | 0.201 | 0.76 (0.39-1.46) | 0.409 |
| | ≥ 48 | 369 | 1.12 (0.57-2.23) | 0.741 | 1.28 (0.62-2.63) | 0.502 |
| Sex | Male | 494 | | | | |
| | Female | 498 | 0.94 (0.57-1.57) | 0.822 | 0.96 (0.57-1.61) | 0.874 |
| Ethnicity | Mandinka | 455 | | | | |
| | Jola | 257 | 0.68 (0.38-1.24) | 0.207 | | |
| | Other | 280 | 0.94 (0.50-1.76) | 0.841 | | |
| Height-for-age z score | > -2 | 796 | | | | |
| | ≤ -2 | 196 | 0.79 (0.43-1.45) | 0.449 | | |
| Prior number of OPV doses | 0-5 | 111 | | | | |
| | 6-7 | 636 | 0.27 (0.06-1.12) | 0.071 | 0.27 (0.06-1.15) | 0.076 |
| | 8-10 | 245 | 0.20 (0.05-0.85) | 0.029 | 0.20 (0.05-0.91) | 0.038 |
| Designation of vaccinator | Nurse | 576 | | | | |
| | PHO | 416 | 0.66 (0.40-1.10) | 0.109 | | |
| Time taken to vaccinate (minutes) | < 1 | 431 | | | | |
| | 1 - <2 | 537 | 0.85 (0.50-1.45) | 0.551 | 0.73 (0.42-1.26) | 0.261 |
| | ≥ 2 | 24 | 0.43 (0.12-1.53) | 0.191 | 0.25 (0.07-0.97) | 0.045 |
| Maternal education§ | No school | 475 | | | | |
| | 1-9 years | 339 | 0.86 (0.49-1.52) | 0.601 | | |
| | >9 years | 178 | 0.79 (0.40-1.57) | 0.501 | | |
| Maternal occupation‡ | At home | 692 | | | | |
| | Small trade | 224 | 2.24 (1.05-4.79) | 0.037 | 2.20 (1.00-4.85) | 0.049 |
| | Profession | 76 | 2.02 (0.62-6.62) | 0.247 | 2.02 (0.60-6.77) | 0.254 |
| Baseline seropositivity‖ | Negative | 53 | | | | |
| | Positive | 939 | | | | |
| ICC | Team: 0.003 | | | | | |

N&S – needle and syringe; IDA – intradermal adapter; DSJI – disposable syringe jet injector; PHO – public health officer; OPV – oral poliovirus vaccine; OR – odds ratio; CI – confidence interval; ICC – Intraclass correlation coefficient § 1-9 years: lower and upper basic school->9 years: secondary and/or college education; ‡ small trade: small trader/unskilled worker-profession: professional and other skilled work; ‖ Baseline seropositivity was not included in the regression because all the 53 individuals that were seronegative at baseline had an IR

**Table S7: Multilevel logistic regression model examining the associations with serotype 3 immune response rates allowing for clustering within vaccination teams.**

| Serotype 3 | | | Unadjusted | | Adjusted | |
|---|---|---|---|---|---|---|
| | | N | OR (95% CI) | p-value | OR (95% CI) | p-value |
| Administration method | N&S | 433 | | | | |
| | IDA | 411 | 0.55 (0.21-1.40) | 0.21 | 0.45 (0.17-1.19) | 0.107 |
| | DSJI | 448 | 0.38 (0.18-0.81) | 0.012 | 0.44 (0.21-0.92) | 0.028 |
| Age (months) | 24 - <36 | 399 | | | | |
| | 36 - <48 | 444 | 1.20 (0.72-2.01) | 0.486 | 1.23 (0.72-2.09) | 0.445 |
| | ≥ 48 | 449 | 1.87 (1.05-3.33) | 0.034 | 1.84 (1.02-3.32) | 0.044 |
| Sex | Male | 634 | | | | |
| | Female | 658 | 1.05 (0.68-1.65) | 0.815 | 1.16 (0.73-1.84) | 0.532 |
| Ethnicity | Mandinka | 618 | | | | |
| | Jola | 331 | 1.36 (0.73-2.55) | 0.33 | | |
| | Other | 343 | 1.12 (0.65-1.94) | 0.674 | | |
| Height-for-age z score | > -2 | 1032 | | | | |
| | ≤ -2 | 260 | 0.73 (0.43-1.24) | 0.243 | | |
| Prior number of OPV doses | 0-5 | 152 | | | | |
| | 6-7 | 858 | 1.19 (0.63-2.25) | 0.589 | | |
| | 8-10 | 282 | 1.95 (0.86-4.43) | 0.111 | | |
| Designation of vaccinator | Nurse | 717 | | | | |
| | PHO | 575 | 0.62 (0.32-1.20) | 0.156 | | |
| Time taken to vaccinate (minutes) | < 1 | 566 | | | | |
| | 1 - <2 | 692 | 1.12 (0.71-1.77) | 0.635 | | |
| | ≥ 2 | 34 | 1.41 (0.32-6.24) | 0.648 | | |
| Maternal education§ | No school | 616 | | | | |
| | 1-9 years | 418 | 1.02 (0.61-1.68) | 0.95 | | |
| | >9 years | 258 | 1.24 (0.66-2.31) | 0.501 | | |
| Maternal occupation‡ | At home | 915 | | | | |
| | Small trade | 280 | 0.93 (0.54-1.58) | 0.776 | | |
| | Profession | 97 | 0.97 (0.40-2.33) | 0.94 | | |
| Baseline seropositivity | Negative | 243 | | | | |
| | Positive | 1049 | 5.22 (3.28-8.29) | <0.001 | 5.20 (3.27-8.28) | <0.001 |
| ICC | Team: 0.012 | | | | | |

N&S – needle and syringe; IDA – intradermal adapter; DSJI – disposable syringe jet injector; PHO – public health officer; OPV – oral poliovirus vaccine; OR – odds ratio; CI – confidence interval; ICC – Intraclass correlation coefficient; § 1-9 years: lower and upper basic school->9 years: secondary and/or college education; ‡ small trade: small trader/unskilled worker-profession: professional and other skilled work

**Table S8: Multilevel logistic regression examining associations with immune response allowing for clustering within vaccination teams and within subjects within teams.**

| | | | Unadjusted | | Adjusted | |
|---|---|---|---|---|---|---|
| **Factor** | **Category** | **N** | **OR (95% CI)** | **p-value** | **OR (95% CI)** | **p-value** |
| Administration method | N&S | 299 | | | | |
| | IDA | 320 | 1.07 (0.47-2.44) | 0.879 | 0.98 (0.44-2.20) | 0.961 |
| | DSJI | 288 | 0.35 (0.18-0.69) | 0.002 | 0.36 (0.18-0.71) | 0.047 |
| Serotype* | 1 | 907 | | | | |
| | 2 | 992 | 0.74 (0.47-1.18) | 0.208 | 0.63 (0.39-1.00) | 0.051 |
| | 3 | 1292 | 0.71 (0.46-1.10) | 0.127 | 0.85 (0.54-1.32) | 0.465 |
| Baseline seropositivity | Negative | 103 | | | | |
| | Positive | 804 | 4.12 (2.56-6.62) | <0.001 | 4.31 (2.63-7.04) | <0.001 |
| Age (months) | 24 - <36 | 270 | | | | |
| | 36 - <48 | 316 | 0.99 (0.59-1.64) | 0.955 | 1.00 (0.60-1.67) | 0.996 |
| | ≥ 48 | 321 | 1.74 (1.01-3.01) | 0.048 | 1.67 (0.97-2.89) | 0.066 |
| Sex | Male | 455 | | | | |
| | Female | 452 | 0.98 (0.64-1.51) | 0.923 | | |
| Ethnicity | Mandinka | 406 | | | | |
| | Jola | 247 | 0.93 (0.52-1.64) | 0.793 | | |
| | Other | 254 | 0.97 (0.56-1.66) | 0.902 | | |
| Height-for-age z score | > -2 | 712 | | | | |
| | ≤ -2 | 195 | 0.80 (0.47-1.35) | 0.397 | | |
| Prior number of OPV doses | 0-5 | 108 | | | | |
| | 6-7 | 600 | 1.11 (0.57-2.17) | 0.767 | | |
| | 8-10 | 199 | 1.08 (0.50-2.33) | 0.849 | | |
| Designation of vaccinator | Nurse | 529 | | | | |
| | PHO | 378 | 0.70 (0.40-1.23) | 0.213 | | |
| Time taken to vaccinate (minutes) | < 1 | 393 | | | | |
| | 1 - <2 | 494 | 1.05 (0.67-1.64) | 0.820 | | |
| | ≥ 2 | 20 | 0.75 (0.20-2.82) | 0.672 | | |
| Maternal education | No schooling | 430 | | | | |
| | 1-9 years: lower/ upper basic | 314 | 0.98 (0.60-1.60) | 0.934 | | |
| | >9 years: secondary/ college | 163 | 0.91 (0.51-1.65) | 0.764 | | |
| Maternal occupation | At home/ housewife | 650 | | | | |
| | Small trader/non-skilled | 188 | 1.13 (0.66-1.96) | 0.654 | | |
| | Professional and other | 69 | 1.04 (0.45-2.40) | 0.921 | | |
| ICC | Team: 0.03 | | | | | |
| | Participants within teams: 0.52 | | | | | |

N&S – needle and syringe; IDA – intradermal adapter; DSJI – disposable syringe jet injector; PHO – public health officer; OPV – oral poliovirus vaccine; OR – odds ratio; CI – confidence interval; ICC – Intraclass correlation coefficient;*Serotype is repeated per individual while other variables appear once for every individual; Team - Correlation between observations within the same vaccination team; Study id| Team – Correlation between observations within the same subject and team.

## Table S9: Logistic regression model examining the association between markers of injection quality and immune response to ID fIPV

| | Unadjusted | | Adjusted‡ | | Adjusted§ | |
|---|---|---|---|---|---|---|
| | OR (95% CI) | p-value | OR (95% CI) | p-value | OR (95% CI) | p-value |
| **Serotype 1** | | | | | | |
| ID fluid bleb size (mm) | 1.12 (1.00-1.26) | 0.053 | 1.15 (1.01-1.31) | 0.039 | 1.09 (0.94-1.27) | 0.251 |
| Fluid loss onto skin (μL) | | | | | | |
| Dry | 1.00 | 0.841 | 1.00 | 0.873 | 1.00 | 0.910 |
| <5 | 1.03 (0.53-2.01) | | 0.99 (0.49-1.97) | | 0.94 (0.47-1.91) | |
| 5 - <10 | 0.93 (0.21-4.16) | | 0.74 (0.16-3.54) | | 0.90 (0.18-4.41) | |
| 10 - <20 | 0.50 (0.14-1.79) | | 0.50 (0.13-1.90) | | 0.57 (0.14-2.23) | |
| ≥20 | 1.26 (0.28-5.61) | | 1.09 (0.22-5.28) | | 1.42 (0.28-7.14) | |
| Good quality injection‖ | | | | | | |
| No | 1.00 | 0.043 | 1.00 | 0.054 | 1.00 | 0.312 |
| Yes | 1.87 (1.02-3.43) | | 1.89 (0.99-3.62) | | 1.47 (0.70-3.08) | |
| **Serotype 2** | | | | | | |
| ID fluid bleb size (mm) | 1.13 (1.02-1.24) | 0.019 | 1.13 (1.02-1.26) | 0.020 | 1.11 (0.97-1.25) | 0.118 |
| Fluid loss onto skin (μL) | | | | | | |
| Dry | 1.00 | 0.232 | 1.00 | 0.252 | 1.00 | 0.297 |
| <5 | 1.21 (0.68-2.16) | | 1.16 (0.64-2.11) | | 1.27 (0.69-2.34) | |
| 5 - <10 | 1.67 (0.38-7.28) | | 2.06 (0.46-9.32) | | 2.73 (0.60-12.5) | |
| 10 - <20 | 0.37 (0.13-1.04) | | 0.38 (0.13-1.12) | | 0.58 (0.19-1.76) | |
| ≥20 | 1.31 (0.38-4.46) | | 1.48 (0.41-5.37) | | 2.24 (0.61-8.28) | |
| Good quality injection‖ | | | | | | |
| No | 1.00 | 0.006 | 1.00 | 0.006 | 1.00 | 0.048 |
| Yes | 2.07 (1.23-3.49) | | 2.17 (1.24-3.78) | | 1.95 (1.00-3.79) | |
| **Serotype 3** | | | | | | |
| ID fluid bleb size (mm) | 1.09 (1.00-1.19) | 0.040 | 1.06 (0.97-1.17) | 0.203 | 1.02 (0.92-1.14) | 0.687 |
| Fluid loss onto skin (μL) | | | | | | |
| Dry | 1.00 | | 1.00 | | 1.00 | |
| <5 | 1.07 (0.64-1.81) | 0.429 | 0.98 (0.57-1.69) | 0.374 | 0.85 (0.49-1.47) | 0.386 |
| 5 - <10 | 0.63 (0.23-1.69) | | 0.57 (0.20-1.66) | | 0.50 (0.17-1.50) | |
| 10 - <20 | 0.56 (0.19-1.69) | | 0.48 (0.15-1.53) | | 0.42 (0.13-1.40) | |
| ≥20 | 0.59 (0.26-1.33) | | 0.50 (0.20-1.19) | | 0.45 (0.17-1.19) | |
| Good quality injection‖ | | | | | | |
| No | 1.00 | 0.016 | 1.00 | 0.044 | 1.00 | 0.178 |
| Yes | 1.74 (1.11-2.72) | | 1.66 (1.01-2.73) | | 1.47 (0.84-2.56) | |

**Supplementary Tables**

‖ Fluid loss onto the skin of <10 µL and bleb size ≥ 5mm; ‡ Adjusted for prior number of OPV doses-height for age-baseline seropositivity-ethnic group-maternal occupation-maternal schooling level-and designation; § Additionally adjusted for administration method

**Table S10: Local and systemic reactogenicity data on day 3 post-vaccination**

| | **N&S (n=915)** | **DSJI (n=920)** | **IDA (n=866)** | **Total (n=2701)** |
|---|---|---|---|---|
| **Tenderness** n/N (%) | | | | |
| No tenderness | 913/915 (99.8%) | 919/920 (99.9%) | 864/866 (99.8%) | 2696/2701 (99.8%) |
| Grade 1 | 2/915 (0.2%) | 1/920 (0.1%) | 2/866 (0.2%) | 5/2701 (0.2%) |
| Grade 2 | 0/915 | 0/920 | 0/866 | 0/2701 |
| Grade 3 | 0/915 | 0/920 | 0/866 | 0/2701 |
| Grade 4 | 0/915 | 0/920 | 0/866 | 0/2701 |
| Any grade 1 – 4 | 2/915 (0.2%) | 1/920 (0.1%) | 2/866 (0.2%) | 5/2701 (0.2%) |
| **Erythema** n/N (%) | | | | |
| No erythema | 914/915 (99.9%) | 920/920 (100.0%) | 866/866 (100.0%) | 2700/2701 (100.0%) |
| Grade 1 | 1/915 (0.1%) | 0/920 | 0/866 | 1/2701(<0.1%) |
| Grade 2 | 0/915 | 0/920 | 0/866 | 0/2701 |
| Grade 3 | 0/915 | 0/920 | 0/866 | 0/2701 |
| Grade 4 | 0/915 | 0/920 | 0/866 | 0/2701 |
| Any grade 1 – 4 | 1/915 (0.1%) | 0/920 | 0/866 | 1/2701 (<0.1%) |
| Mean erythema (mm) in those with any erythema | 2.5 | - | - | 2.5 |
| **Induration** n/N (%) | | | | |
| No induration | 912/915 (99.8%) | 915/920 (99.5%) | 866/866 (100.0%) | 2693/2701 (99.7%) |
| Grade 1 | 2/915 (0.2%) | 4/920 (0.4%) | 0/866 | 6/2701 (0.2%) |
| Grade 2 | 0/915 | 1/920 (0.1%) | 0/866 | 1/2701 (<0.1%) |
| Grade 3 | 0/915 | 0/920 | 0/866 | 0/2701 |
| Grade 4 | 0/915 | 0/920 | 0/866 | 0/2701 |
| Any grade 1 – 4 | 2/915 (0.2%) | 5/920 (0.5%) | 0/866 | 7/2701 (0.3%) |
| Mean induration in those with any induration | 1.75 (0 - 11.28) | 1.68 (0 - 4.15) | - | 1.70 (0.34 - 3.06) |

**Supplementary Tables**

| | **N&S (n=915)** | **DSJI (n=920)** | **IDA (n=866)** | **Total (n=2701)** |
|---|---|---|---|---|
| **Temperature** (ºC) | | | | |
| Mean (95% CI) | 36.0  (36.0 – 36.0) | 36.1 (36.0 - 36.1) | 36.1 (36.0 - 36.1) | 36.0 (36.0 - 36.1) |
| $\leq$ 37.4ºC | 911/915 (99.6%) | 916/920 (99.6%) | 857/866 (99.1%) | 2684/2701 (99.4%) |
| 37.5 – 38.0ºC | 3/915 (0.3%) | 2/920 (0.2%) | 6/866 (0.7%) | 11/2701 (0.4%) |
| 38.1 – 39.0ºC | 1/915 (0.1%) | 2/920 (0.2%) | 2/866 (0.2%) | 5/2701 (0.2%) |
| > 39.0ºC | 0/915 | 0/920 | 0/866 | 0/2701 |
| **Vomiting** n/N (%) | | | | |
| No vomiting | 907/915 (99.1%) | 909/920 (98.8%) | 851/866 (98.3%) | 2667/2701 (98.7%) |
| Grade 1 | 6/915 (0.7%) | 7/920 (0.8%) | 10/866 (1.2%) | 23/2701 (0.9%) |
| Grade 2 | 2/915 (0.2%) | 3/920 (0.3%) | 5/866 (0.6%) | 10/2701 (0.4%) |
| Grade 3 | 0/915 | 1/920 (0.1%) | 0/866 | 1/2701 (<0.1%) |
| Grade 4 | 0/915 | 0/920 | 0/866 | 0/2701 |
| Any grade 1 – 4 | 8/915 (0.9%) | 11/920 (1.2%) | 15/866 (1.7%) | 34/2701 (1.3%) |
| **Diarrhoea** n/N (%) | | | | |
| No diarrhoea | 883/915 (96.5%) | 873/920 (94.9%) | 835/866 (96.4%) | 2591/2701 (95.9%) |
| Grade 1 | 28/915 (3.1%) | 42/920 (4.6%) | 29/866 (3.4%) | 99/2701 (3.7%) |
| Grade 2 | 4/915 (0.4%) | 4/920 (0.4%) | 2/866 (0.2%) | 10/2701 (0.4%) |
| Grade 3 | 0/915 | 1/920 (0.1%) | 0/866 | 1/2701 (<0.1%) |
| Grade 4 | 0/915 | 0/920 | 0/866 | 0/2701 |
| Any grade 1 – 4 | 32/915 (3.5%) | 47/920 (5.1%) | 31/866 (3.6%) | 110/2701 (4.1%) |

**Supplementary Tables**

| | **N&S (n=915)** | **DSJI (n=920)** | **IDA (n=866)** | **Total (n=2701)** |
|---|---|---|---|---|
| **Feeding** n/N (%) | | | | |
| Feeding normally | 904/915 (98.8%) | 905/920 (98.4%) | 853/866 (98.5%) | 2662/2701 (98.6%) |
| Grade 1 | 12/915 (1.4%) | 15/920 (1.6%) | 12/866 (1.4%) | 38/2701 (1.4%) |
| Grade 2 | 0/915 | 0/920 | 0/866 | 0/2701 |
| Grade 3 | 1/915 (0.1%) | 0/920 | 1/866 (0.1%) | 1/2701 (<0.1%) |
| Grade 4 | 0/915 | 0/920 | 0/866 | 0/2701 |
| Any grade 1 – 4 | 13/915 (1.2%) | 15/920 (1.6%) | 13/866 (1.5%) | 39/2701 (1.4%) |
| **Drowsiness** n/N (%) | | | | |
| No drowsiness | 915/915 (100.0%) | 912/920 (99.1%) | 861/866 (99.4%) | 2688/2701 (99.5%) |
| Grade 1 | 0/915 | 7/920 (0.8%) | 5/866 (0.6%) | 12/2701 (0.4%) |
| Grade 2 | 0/915 | 1/920 (0.1%) | 0/866 | 1/2701 (<0.1%) |
| Grade 3 | 0/915 | 0/920 | 0/866 | 0/2701 (<0.1%) |
| Grade 4 | 0/915 | 0/920 | 0/866 | 0/2701 |
| Any grade 1 – 4 | 0/915 | 8/920 (0.9%) | 5/866 (0.6%) | 13/2701 (0.5%) |
| **Irritability** n/N (%) | | | | |
| No irritability | 913/915 (99.8%) | 914/920 (99.4%) | 861/866 (99.4%) | 2688/2701 (99.5%) |
| Grade 1 | 2/915 (0.2%) | 6/920 (0.7%) | 3/866 (0.4%) | 11/2701 (0.4%) |
| Grade 2 | 0/915 | 0/920 | 2/866 (0.2%) | 2/2701 (0.1%) |
| Grade 3 | 0/915 | 0/920 | 0/866 | 0/2701 |
| Grade 4 | 0/915 | 0/920 | 0/866 | 0/2701 |
| Any grade 1 – 4 | 2/915 (0.2%) | 6/920 (0.7%) | 5/866 (0.6%) | 13/2701 (0.5%) |

N&S – needle and syringe; IDA – intradermal adapter; DSJI – disposable syringe jet injector

**Table S11: Adverse events**

| | | N&S (n=917) | DSJI (n=929) | IDA (n=874) | Total (n=2720) |
|---|---|---|---|---|---|
| Blood and Lymphatic system disorders | Any AE-n/N (%) | 4/917 (0.4%) | 2/929 (0.2%) | 1/874 (0.1%) | 7/2720 (0.3%) |
| | Severe (grade 3)-AE n/N (%) | 0/917 (0.0%) | 0/929 (0.0%) | 0/874 (0.0%) | 0/2720 (0.0%) |
| Ear and labyrinth disorders | Any AE-n/N (%) | 1/917 (0.1%) | 1/929 (0.1%) | 0/874 (0.0%) | 2/2720 (0.1%) |
| | Severe (grade 3)-AE n/N (%) | 0/917 (0.0%) | 0/929 (0.0%) | 0/874 (0.0%) | 0/2720 (0.0%) |
| Gastrointestinal disorders | Any AE-n/N (%) | 40/917 (4.4%) | 55/929 (5.9%) | 42/874 (4.8%) | 137/2720 (5.0%) |
| | Severe (grade 3)-AE n/N (%) | 0/917 (0.0%) | 0/929 (0.0%) | 0/874 (0.0%) | 0/2720 (0.0%) |
| General disorders and administration site conditions | Any AE-n/N (%) | 31/917 (3.4%) | 7/929 (0.8%) | 18/874 (2.1%) | 56/2720 (2.1%) |
| | Severe (grade 3)-AE n/N (%) | 0/917 (0.0%) | 0/929 (0.0%) | 1/874 (0.1%) | 1/2720 (<0.1%) |
| Infections and infestations | Any AE-n/N (%) | 122/917 (13.3%) | 126/929 (13.6%) | 194/874 (22.2%) | 442/2720 (16.3%) |
| | Severe (grade 3)-AE n/N (%) | 2/917 (0.2%) | 0/929 (0.0%) | 1/874 (0.1%) | 3/2720 (0.1%) |
| Injury-poisoning and procedural complications | Any AE-n/N (%) | 9/917 (1.0%) | 1/929 (0.1%) | 7/874 (0.8%) | 17/2720 (0.6%) |
| | Severe (grade 3)-AE n/N (%) | 0/917 (0.0%) | 0/929 (0.0%) | 0/874 (0.0%) | 0/2720 (0.0%) |
| Metabolism and nutrition disorders | Any AE-n/N (%) | 5/917 (0.6%) | 2/929 (0.2%) | 0/874 (0.0%) | 7/2720 (0.3%) |
| | Severe (grade 3)-AE n/N (%) | 1/917 (0.1%) | 0/929 (0.0%) | 0/874 (0.0%) | 1/2720 (<0.1%) |
| Musculoskeletal and connective tissue disorders | Any AE-n/N (%) | 2/917 (0.2%) | 1/929 (0.1%) | 0/874 (0.0%) | 3/2720 (0.1%) |
| | Severe (grade 3)-AE n/N (%) | 0/917 (0.0%) | 0/929 (0.0%) | 0/874 (0.0%) | 0/2720 (0.0%) |
| Respiratory-thoracic and mediastinal disorders | Any AE-n/N (%) | 5/917 (0.6%) | 12/929 (1.3%) | 0/874 (0.0%) | 17/2720 (0.6%) |
| | Severe (grade 3)-AE n/N (%) | 0/917 (0.0%) | 0/929 (0.0%) | 0/874 (0.0%) | 0/2720 (0.0%) |
| Skin and cutaneous tissue disorders | Any AE-n/N (%) | 15/917 (1.6%) | 11/929 (1.2%) | 13/874 (1.5%) | 39/2720 (1.4%) |
| | Severe (grade 3)-AE n/N (%) | 0/917 (0.0%) | 0/929 (0.0%) | 0/874 (0.0%) | 0/2720 (0.0%) |
| Vascular disorders | Any AE-n/N (%) | 0/917 (0.0%) | 1/929 (0.1%) | 0/874 (0.0%) | 1/2720 (<0.1%) |
| | Severe (grade 3)-AE n/N (%) | 0/917 (0.0%) | 0/929 (0.0%) | 0/874 (0.0%) | 0/2720 (0.0%) |
